# The Impact of Electroacupuncture at Hegu, Shousanli, and Quchi Based on the Theory “Treating Flaccid Paralysis by Yangming Alone” on Stroke Patients' EEG: A Pilot Study

**DOI:** 10.1155/2020/8839491

**Published:** 2020-11-24

**Authors:** Fei Zou, Yi-Fang Lin, Shu-Geng Chen, Lei Cao, Hao-ran Wang, Bin Ye, Qiang Wang, He Jie-Ying, Jie Jia

**Affiliations:** ^1^Department of Rehabilitation Medicine, Huashan Hospital, Fudan University, Shanghai, China; ^2^Department of Rehabilitation Medicine, Shanghai Jing'an District Central Hospital, Shanghai, China; ^3^Department of Electronic Engineering, Shanghai Maritime University, Shanghai, China; ^4^Department of Computer Science and Technology, College of Electronic and Information Engineering, Tongji University, Shanghai, China; ^5^Department of Rehabilitation Medicine, The Shanghai Third Rehabilitation Hospital, Shanghai, China

## Abstract

**Background:**

In China, electroacupuncture based on meridians theory “treating flaccid paralysis by *Yangming* alone” has been widely used for stroke rehabilitation in clinical practice. The aim of this study was to explore the electroencephalography change of electroacupuncture intervention on strokes patients with flaccid paralysis.

**Methods:**

Twenty-three stroke patients with flaccid paralysis and six stroke patients with spasticity accepted electroacupuncture with the acupoints Hegu [LI4], Shousanli [LI10], and Quchi [LI11] for 20 minutes and their EEG data were recorded before, during, and after the electroacupuncture intervention.

**Results:**

Compared with the baseline EEG signals before electroacupuncture, the ipsilesional and contralesional beta-band average power of patients with flaccid paralysis and spasticity were significantly increased during the needles retention stage and decreased slightly after removing the needles. The significant decrease of the ipsilesional and contralesional delta band average power in patients with flaccid paralysis occurred during the electroacupuncture stimulation, and they increased after the removal of the needles. The ipsilesional delta band average power of patients with spasticity significantly decreased during the electroacupuncture stimulation.

**Conclusion:**

From this pilot electrophysiological study, we provided a possible electrophysiological mechanism of the curative effect of electroacupuncture for stroke rehabilitation.

## 1. Introduction

Motor impairment of the upper extremity caused by a cerebral vascular accident is quite difficult to recover from [1]. To improve performance in the functional movement of the upper extremity, we have done a lot of work on scientific research and clinical applications such as mirror therapy, motor imagery, brain-computer interface, and other rehabilitation techniques [2–5]. Electroacupuncture (EA) treatment, an effective alternative approach for improving motor impairment of the upper extremity in stroke patients, is becoming an interesting research point, especially for the management of poststroke flaccid paralysis in clinical practice [6–8]. EA based on the meridians theory of Traditional Chinese Medicine (TCM) “treating flaccid paralysis by *Yangming* alone” was one of the most common rehabilitative approaches for apoplexy and it is still applied in clinical treatment nowadays [9]. This classical theory means the acupoints of Large Inte Stine could be used for treating flaccidity syndrome. From the TCM perspective, there is no distinction between flaccid paralysis and spasticity in stroke patients. It thinks that flaccid paralysis and spasticity are the two kinds of different performances of “liver qi” catharsis too much, and the acupoints of the Large Inte Stine and Stomach could be used for rebalancing the “liver qi” [10]. Some literature has demonstrated that the acupoints of Large Inte Stine could enhance handgrip strength and pinch strength, thus improving motor impairment, in stroke patients [11, 12]. Previous studies have shown that Hegu [LI4], Shousanli [LI10], and Quchi [LI11] of Large Inte Stine are frequently used acupoints for the upper extremity motor impairment in stroke patients [8, 13].

With the deepening understanding of stroke rehabilitation and the traditional Chinese medicine theory, the mechanism of EA on stroke patients needs to be discovered. How EA combined with modern rehabilitation can play a better role in clinical practice is a question worthy of thinking about. In this study, we intend to explore the change of brain activity in stroke patients during EA treatment.

Electroencephalography (EEG), with an advantage of high time resolution, can detect real-time cortical electrical activity in the cortex [14]. EEG is widely used in the research of brain-computer interface training, the analysis of functional connectivity and brain network in different crowds, the investigation of brain spectral power as a biomarker on disease or injury, and so on [15, 16]. The different frequency of brain wave oscillations had been regarded as biomarkers of injury or recovery after stroke [17, 18]. Beta-band (13–30 Hz) oscillations are reported to traditionally link to motor functions [19]. Additionally, it also played an essential role in the interactions between the motor cortex and the other cortex, such as auditory and sensorimotor brain areas [20]. Recently, some research pointed out that low-frequency oscillations in the delta band (1–3 Hz) related to the motor recovery of stroke. Cassidy et al. reported that delta band power is associated with greater injury and better motor status in the chronic phase [21]. Bönstrup et al. provided evidence for a link between low-frequency oscillations and functional recovery after stroke [22]. Linear models implied a strong relationship between beta-band activity in frontal, central, and parietal regions with upper extremity motor recovery and suggested that delta band power in the primary motor cortex related to better motor status in the chronic stage [21, 23].

To explore the changes of brain wave oscillation of stroke patients with EA treatment based on the theory “treating flaccid paralysis by *Yangming* alone”, in this study, we propose inserting needles at the Hegu [LI4], Shousanli [LI10], and Quchi [LI11] in the hemiplegic upper extremity of stroke patients with flaccid paralysis and observing brain wave power change with spectrum analysis. Besides, we also recruited 6 stroke patients with spasticity as a control group. We assumed that EA treatment would influence EEG activity, especially the beta rhythm and delta rhythm, which might provide the electrophysiological mechanism of the curative effect of EA for stroke rehabilitation.

## 2. Methods

### 2.1. Study Design and Participants

**Figure 1:**
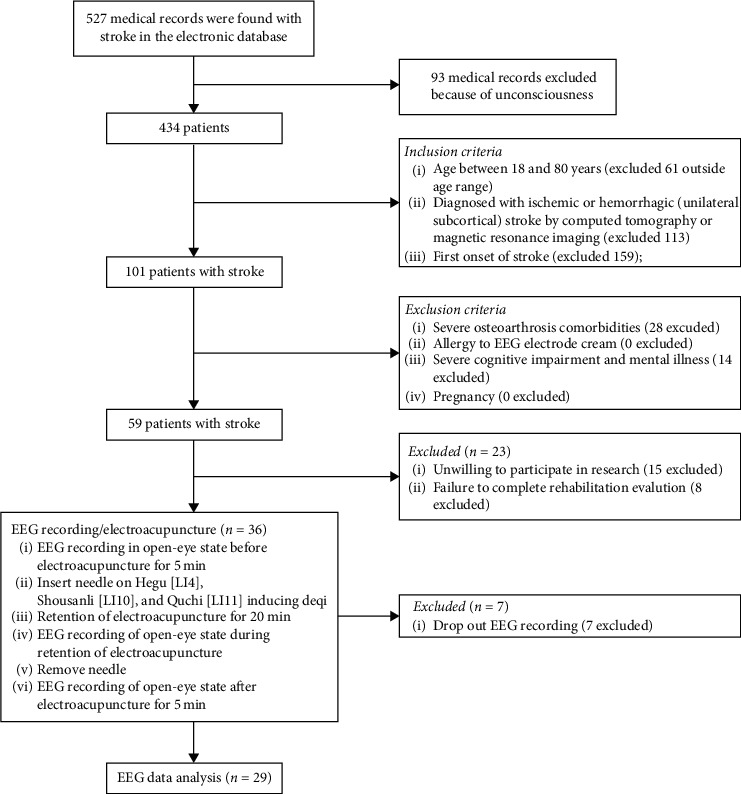
Flow chart of the study participant.

We recruited inpatients with stroke admitted to the Department of Rehabilitation Medicine, Huashan Hospital, and Shanghai Third Rehabilitation Hospital from April 2020 to August 2020. All patients received the rehabilitation assessments, including the Fugl-Meyer assessment Upper Limb subscale (FMA-UL), Barthel index (BI), and National Institute of Health stroke scale (NIHSS). The inclusion criteria were (1) age between 18 and 80 years; (2) diagnosed with ischemic or hemorrhagic (unilateral subcortical) stroke by computed tomography or magnetic resonance imaging; and (3) the first onset of stroke. The exclusion criteria were (1) severe osteoarthrosis comorbidities; (2) allergy to EEG electrode cream; (3) severe cognitive impairment and mental illness; and (4) pregnancy. A total of 29 patients met these criteria and were enrolled in this study. All patients were informed about the electroacupuncture stimulation as follows: “three acupuncture pins will be inserted into the muscle at three different acupoints of the affected upper extremity” and signed the informed consent forms prior to the participation according to the Declaration of Helsinki.  Figure 1 displayed the flow chart of the study subjects. Demographic and clinical characteristics of participants were shown in Table 1. This study was approved by the Medical Ethics Committee of Jing'an District Central Hospital of Shanghai (Ethics reference number: 2020–29), and the trial was registered on the Chinese clinical trial registry (ChiCTR2000036959).

### 2.2. Electroacupuncture Stimulation

**Figure 2:**
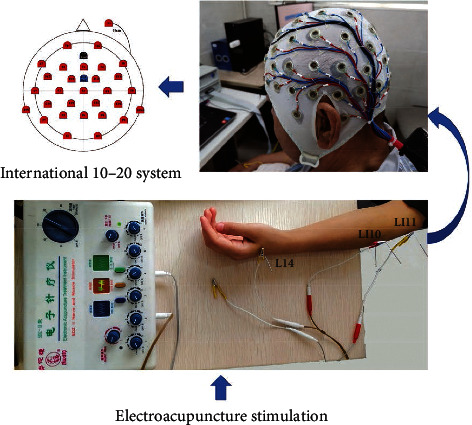
The selected acupoints of the experiment.

An acupuncturist with more than 5 years of clinical experience inserted the sterile disposable acupuncture needles (Jiajian, 0.30 × 40 mm; Wuxi Jiajian Medical Instruments, Wuxi, China) in three acupoints of Large Inte Stine on the hemiplegia side (Hegu [LI4], Shousanli [LI10], and Quchi [LI11]), as shown in Figure 2. These acupoints will be needled perpendicularly, with a depth of 10–15 mm approximately. Following insertion, electrical stimulation was applied to the needles with the intermittent wave, the frequency of 2 Hz, and the current intensity adjusted according to the patients' tolerance [24, 25]. After the 20-minute retention of electroacupuncture, the needles were removed.

### 2.3. EEG Recording

All patients' brain activities were measured by EEG in a sitting position with eyes opened before, during, and after EA treatment. A 32-channel EEG based on the international 10–20 system was placed on the patients' scalp and recorded in a quiet room using BrainCap (Brain Products, Gilching, Germany) with a sampling rate of 1000 Hz. The ground electrode and reference electrode were placed in front and behind the Fz electrode, respectively. The electrode impedances were set to <5 kΩ. The brain wave data were continuously recorded for 30 minutes, including the 5-minute baseline EEG recording before EA treatment, the 20-minute EEG recording during the EA period, and the 5-minute EEG recording after removal of needles.

### 2.4. EEG Processing

The spectrum analysis was used for revealing brain waves activities of 32 channels (FP1, FP2, F3, F4, C3, C4, P3, P4, O1, O2, F7, F8, T7, T8, P7, P8, Fz, Cz, Pz, IO, FC1, FC2, CP1, CP2, FC5, FC6, CP5, CP6, FT9, FT10, TP9, and TP10). Firstly, raw data were band-pass filtered from 0 to 35 Hz. Then, ECG artifacts were removed by using EEGLAB software. After preprocessing, EEGs are divided into the following bands: *δ* (0.5–4 HZ), *θ* (4–8 HZ), *α* (8–13 HZ), and *β* (13–30 HZ). These features were commonly used for evaluating changes in mental activities [25]. Particularly, the signals were divided into 4 pieces averagely for detailed analysis during the electroacupuncture stimulation period. Data for patients with infarct in the damage (right/left) hemisphere was flipped along the midsagittal plane so that the contralesional (left/right) hemisphere corresponded to the ipsilesional hemisphere for all patients. Data for patients with the contralesional hemisphere were also flipped along the midsagittal plane for all patients. We adopted several channels that are associated with motor function for further analysis. They are FC1, FC5, C3, CP1, and CP5 in the left hemisphere and FC2, FC6, C4, CP2, and CP6 in the right hemisphere.

### 2.5. Statistics

The statistical analyses were conducted using SPSS version 25.0 (IBM Inc., Chicago, IL, USA).

**Figure 3:**
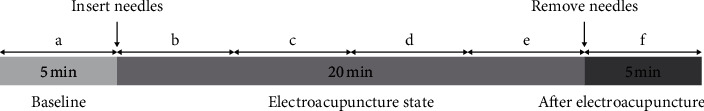
Experimental process of electroacupuncture. (a) The baseline with rest state before electroacupuncture; (b)-(e) During the electroacupuncture with the open-eye state. (f) The rest state after electroacupuncture. (a) Power average of 1–5 minutes. (b)-(e) Power average of every five minutes. (f) Power average of 5 minutes after removing needles.

The average power of 5 minutes before the EA treatment was regarded as the baseline, “a”. During the EA state, the average power was divided into four phases to calculate, of which “b” is the average power between 5 and 10 minutes, “c” between 10 and 15 minutes, “d” between 15 and 20 minutes, and “e” between 20 and 25 minutes. The average power of 5 minutes after removing needles was named “f” (see Figure 3). One-sample Kolmogorov-Smirnov-tests were firstly used to verify whether the variables followed a normal. After this test indicated non-formal distribution. All data are presented with average (mean) ± standard deviation (SD) (see Tables 2 and 3 for details) and was calculated through Generalized estimating equations (GEEs) to study the statistical differences. GEEs, as a statistical analysis approach used for abnormal distributed repeated measures data, was first proposed by Zeger and Liang in 1986 [27]. In this study, the parameter of GEEs is an unstructured correlation structure with robust variance estimation for CIs and *p* values.

## 3. Results

The statistical analysis suggested that the performances of channels FC1, FC5, CP1, and CP5 were similar to channel C3. Channels FC2, FC6, CP2, and CP6 were similar to channel C4. In this study, channels C3 and C4 are representative of motor areas.

**Figure 4:**
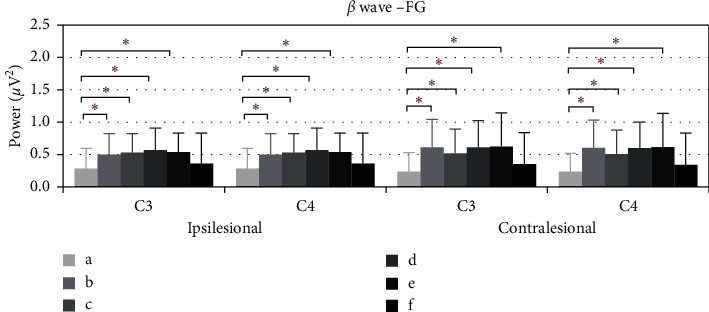
The *β* wave average power of FG of C3 and C4, ^*∗*^*p* < 0.05. (a) Power average of 1–5 minutes. (b)–(e) Power average of every five minutes. (f) Power average of 5 minutes after removing needles.

Figure 4 shows the average power of *β* during the electroacupuncture process of flaccid paralysis subjects. Compared with baseline, the average power of *β* in ipsilesional C3 rose at 5–10 minutes (*p*=0.021), 10–15 minutes (*p*=0.07), 15–20 minutes (*p*=0.001), and 20–25 minutes (*p*=0.04), as well as at the ipsilesional C4. Upon removing the needles, the power decreased, and no significant differences are noted. As for the contralesional C3, the average power of *β* rose at 5–10 minutes (*p*=0.001), 10–15 minutes (*p*=0.05), 15–20 minutes (*p* < 0.001), and 20–25 minutes (*p*=0.02), as well as contralesional C4. Upon removing the needles, no significant difference is noted.

**Figure 5:**
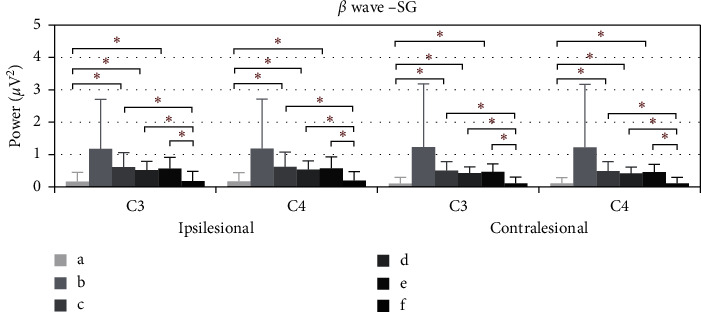
The *β* wave average power of SG of C3 and C4, ^*∗*^*p* < 0.05. (a) Power average of 1–5 minutes. (b)–(e) Power average of every five minutes. (f) Power average of 5 minutes after removing needles.

Figure 5 shows the average power of *β* during the electroacupuncture process in spasticity subjects. Compared with baseline, the average power of *β* in ipsilesional C3 rose at 10–15 minutes (*p*=0.032), 15–20 minutes (*p*=0.005), and 20–25 minutes (*p*=0.016), as well as ipsilesional C4. After removing the needles, the average power decreased and the significant differences are noted compared with those at 10–15 minutes (*p*=0.045), 15–20 minutes (*p*=0.011), and 20–25 minutes (*p*=0.027). As for the contralesional C3, the average power of *β* rose at 10–15 minutes (*p*=0.009), 1520 minutes (*p*=0.002), and 20–25 minutes (*p*=0.008) was compared with baseline, as well as contralesional C4. After removing the needles, the average power abated and significant differences are noted at 10–15 minutes (*p*=0.011), 15–20 minutes (*p*=0.002), and 20–25 minutes (*p*=0.010).

**Figure 6:**
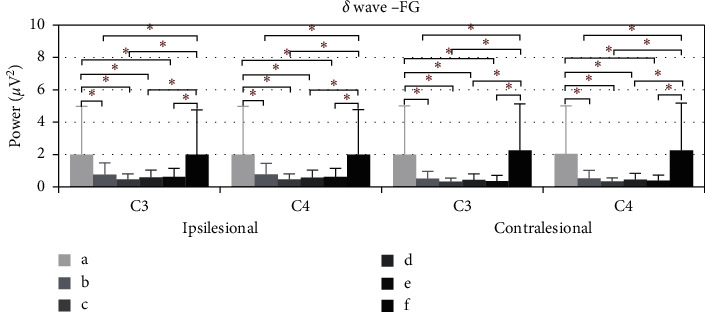
The *δ* wave average power of FG of C3 and C4, ^*∗*^*p* < 0.05. (a) Power average of 1–5 minutes. (b)–(e) Power average of every five minutes. (f) Power average of 5 minutes after removing needles.

Figure 6 shows the average power of *δ* during the electroacupuncture process of flaccid paralysis subjects. Compared with baseline, the average power of *δ* ipsilesional C3 fell at 5–10 minutes (*p*=0.026), 10–15 minutes (*p*=0.010), 15–20 minutes (*p*=0.018), and 20–25 minutes (*p*=0.017), as well as at ipsilesional C4. After removing the needles, the average power rose, and the significant differences are noted at 5–10 minutes (*p*=0.017), 10–15 minutes (*p*=0.006), 15–20 minutes (*p*=0.010), and 20–25 minutes (*p*=0.008). Also, contralesional C3 fell at 5–10 minutes (*p*=0.007), 10–15 minutes (*p*=0.003), 15–20 minutes (*p*=0.005), and 20–25 minutes (*p*=0.003), as well as contralesional C4. After removing the needles, the average power rose, and significant differences are noted at 5–10 minutes (*p*=0.003), 10–15 minutes (*p*=0.001), 15–20 minutes (*p*=0.002), and 20–25 minutes (*p*=0.001).

**Figure 7:**
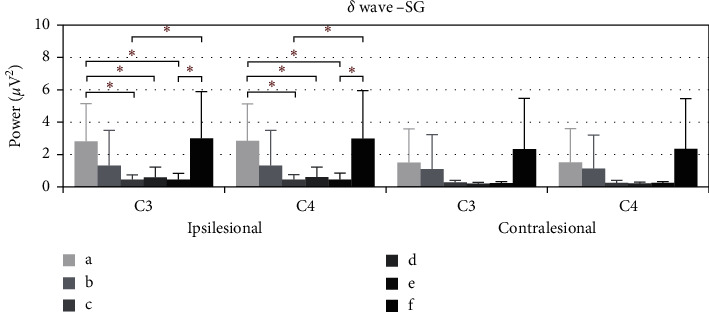
The *δ* wave average power of SG of C3 and C4, ^*∗*^*p* < 0.05. (a) Power average of 1–5 minutes. (b)–(e) Power average of every five minutes. (f) Power average of 5 minutes after removing needles.

Figure 7 shows the average power of *δ* during the electroacupuncture process of spasticity subjects. Compared with baseline, the average power of *δ* in ipsilesional C3 fell at 10–15 minutes (*p*=0.010), 15–20 minutes (*p*=0.034), and 20–25 minutes (*p*=0.013), as well as ipsilesional C4. After removing the needles, the average power rose, and the significant differences are noted at 10–15 minutes (*p*=0.023) and 20–25 minutes (*p*=0.027). Although contralesional C3 and C4 fell at each stage during the electroacupuncture process, no significant difference is noted.

## 4. Discussion

This is a pilot electrophysiological study of electroacupuncture therapy in patients with cerebral vascular accidents. We explored the cortical effects of EA treatment based on the acupuncture theory “treating flaccid paralysis by *Yangming* alone” on beta and delta band oscillations before, during, and after the EA intervention in stroke patients.

In the current study, EA treatment was found to induce modulation of beta and delta band power from the ipsilesional and contralesional primary motor cortex of stroke patients while, in our previous study, we found that Jin's three-needle acupuncture therapy (an empiric treatment) could induce alpha rhythm oscillations from the occipital and parietal areas [28]. As we know, beta-band power oscillations within the primary motor cortex (M1) were reported to be linked to upper limb motor recovery in many studies [19, 29, 30]. Previous research also suggested delta band power in M1 was related to a better motor status [21, 31]. For this reason, we measured C3 and C4 electrodes and analyzed the changes in beta and delta band power.

In Tables 2 and 3, the brain wave average power before the needles are inserted shows that the average power of the delta wave was higher than beta waves. This result is consistent with the conclusion of Rabiller et al., who reported that the lower frequencies (delta and theta) increased and faster frequencies (alpha and beta) decreased after the stroke onset [17]. In another study with healthy subjects, they pointed out that the powers of *α* and *β* waves were stronger than *δ* and *θ* waves [32]. Therefore, compared to healthy individuals, beta oscillations decreased and delta oscillations increased in stroke patients.

In Figures 4 and 6, the ipsilesional and contralesional beta waves average power of stroke patients with flaccid paralysis increased and delta waves average power decreased during the electroacupuncture period. Three possible explanations for this phenomenon are detailed as follows. Firstly, electroacupuncture according to the classic theory adjusted the beta and delta band oscillations from the ipsilesional hemisphere in the direction of a healthy state. Rabiller et al. reported that delta band power increased and beta-band power decreased after stroke onset [17]. Secondly, electroacupuncture also activates the contralesional motor cortex. Park et al. have identified that the beta-band power in the contralesional motor cortex significantly correlated with the motor recovery rates [33]. Thirdly, the brain activities between the hemispheres are interconnecting [34]. The beta and delta band oscillations induced by electroacupuncture stimulation are not limited to the ipsilesional brain. Hence, further exploration is needed for brain network analysis.

In Figures 5 and 7, the performance of the ipsilesional and contralesional beta and delta waves average power of stroke patients with spasticity is similar to the patients with flaccid paralysis. The possible explanation is the readjustment of brain wave activities induced by EA for patients with spasticity. However, Kaiser et al. found that stronger ERD was associated with higher spasticity, and overdrive of the motor system might exist in patients with spasticity [35]. Thus, the beta-band power should be decreased during the electroacupuncture period, while our findings suggested otherwise. Small sample size on spasticity may be the reason for such results.

Our study suggested that electroacupuncture based on “treating flaccid paralysis by *Yangming* alone” induced an increase of beta-band power and a decrease of delta band power in the ipsilesional and contralesional hemispheres during the electroacupuncture needle retention stage. However, as a cross-sectional study, we cannot ensure that the patients will have a similar performance to beta and delta band power oscillation after a period of electroacupuncture treatment. Results from this research could not explain the question of how EA treatment could affect the brain network in the poststroke brain.

The limitations of our study included a small sample size on stroke patients with spasticity and no EEG data collected from healthy individuals and the sham EA group. This may limit the further exploration of brain wave changes in those groups of patients. Based on this pilot study, a further study with a larger sample size could be performed to contribute a clearer result.

## 5. Conclusions

This study demonstrated that the beta-band power increased and delta band power decreased in the bilateral motor cortex during the electroacupuncture treatment. We speculate that increased beta wave and decreased delta wave by electroacupuncture based on the classic theory provides a possible electrophysiological mechanism of the curative effect of electroacupuncture for stroke rehabilitation.

## Figures and Tables

**Table 1:** Demographic and clinical characteristics of participants.

Characteristics	Flaccid paralysis group (*n* = 23)	Spasticity group (*n* = 6)
Age, years, mean (SD)	60.22 (8.974)	62.62 (12.86)
Days after stroke onset [M (QL, QU)]	22 (15, 60)	77.5 (31, 378.5)
NIHSS	7.13 (4.104)	10.50 (9.63)

Gender, *N*		
Male	13	3
Female	10	3

Side of paralysis		
Left	11	4
Right	12	2

Type of stroke		
Ischemic	18	2
Hemorrhagic	5	4

Handedness		
Left	2	0
Right	21	6

**Table 2:** The brain wave power of EEG in the flaccid paralysis group.

	Flaccid paralysis group					
	a	b	c	d	e	f
C3 ipsilesional						
*β*	0.29 (0.31)	0.50 (0.32)^*∗*^	0.53 (0.30)^*∗*^	0.58 (0.34)^*∗*^	0.54 (0.30)^*∗*^	0.37 (0.47)
*δ*	2.02 (2.93)	0.76 (0.70)^*∗*#^	0.45 (0.34)^*∗*#^	0.56 (0.45)^*∗*#^	0.59 (0.54)^*∗*#^	2.03 (2.72)
C4 ipsilesional						
*β*	0.29 (0.31)	0.50 (0.32)^*∗*^	0.53 (0.30)^*∗*^	0.58 (0.34)^*∗*^	0.54 (0.30)^*∗*^	0.37 (0.47)
*δ*	2.02 (2.93)	0.76 (0.70)^*∗*#^	0.45 (0.34)^*∗*#^	0.56 (0.45)^*∗*#^	0.59 (0.54)^*∗*#^	2.03 (2.72)
C3 contralesional						
*β*	0.24 (0.29)	0.62 (0.43)^*∗*^	0.52 (0.37)^*∗*^	0.61 (0.41)^*∗*^	0.63 (0.52)^*∗*^	0.35 (0.49)
*δ*	2.06 (2.92)	0.53 (0.46)^*∗*#^	0.34 (0.23)^*∗*#^	0.43 (0.38)^*∗*#^	0.38 (0.35)^*∗*#^	2.26 (2.90)
C4 contralesional						
*β*	0.24 (0.29)	0.62 (0.43)^*∗*^	0.52 (0.37)^*∗*^	0.61 (0.41)^*∗*^	0.63 (0.52)^*∗*^	0.35 (0.49)
*δ*	2.06 (2.92)	0.53 (0.46)^*∗*#^	0.34 (0.23)^*∗*#^	0.43 (0.38)^*∗*#^	0.38 (0.35)^*∗*#^	2.26 (2.90)

Mean (SD), ^*∗*^significant difference from *a*, ^#^significant difference from *f* (*p* < 0.05).

**Table 3:** The brain wave power of EEG in the spasticity group.

	Spasticity group					
	a	b	c	d	e	f
C3 ipsilesional						
*β*	0.18 (0.28)	1.20 (1.53)	0.64 (0.44)^*∗*^^#^	0.55 (0.27)^*∗*^^#^	0.58 (0.36)^*∗*^^#^	0.19 (0.30)
*δ*	2.88 (2.32)	1.35 (2.17)	0.47 (0.32)^*∗*^^#^	0.63 (0.63)^*∗*^	0.47 (0.41)^*∗*^^#^	3.04 (2.91)
C4 ipsilesional						
*β*	0.18 (0.28)	1.20 (1.53)	0.64 (0.44)^*∗*^^#^	0.55 (0.27)^*∗*^^#^	0.58 (0.36)^*∗*^^#^	0.19 (0.30)
*δ*	2.88 (2.32)	1.35 (2.17)	0.47 (0.32)^*∗*^^#^	0.63 (0.63)^*∗*^	0.47 (0.41)^*∗*^^#^	3.04 (2.91)
C3 contralesional						
*β*	0.12 (0.18)	1.25 (1.94)	0.51 (0.29)^*∗*^^#^	0.44 (0.19)^*∗*^^#^	0.48 (0.25)^*∗*^^#^	0.12 (0.19)
*δ*	1.56 (2.08)	1.17 (2.10)	0.33 (0.13)	0.28 (0.05)	0.31 (0.07)	2.40 (3.10)
C4 contralesional						
*β*	0.12 (0.18)	1.25 (1.94)	0.51 (0.29)^*∗*^^#^	0.44 (0.19)^*∗*^^#^	0.48 (0.25)^*∗*^^#^	0.12 (0.19)
*δ*	1.56 (2.08)	1.17 (2.10)	0.33 (0.13)	0.28 (0.05)	0.31 (0.07)	2.40 (3.10)

Mean (SD), ^*∗*^significant difference from *a*, ^#^significant difference from *f* (*p* < 0.05).

## Data Availability

The data used to support the findings of this research are available from the corresponding author.
